# How Parents Perceive Children’s Values in Indonesian Families: A Multidimensional and Contextual Analysis

**DOI:** 10.12688/f1000research.176108.1

**Published:** 2026-01-23

**Authors:** Yanti Karmila Nengsih, Elih Sudiapermana, Uyu Wahyudin, Joni Rahmat Pramudia, Vina Amilia Suganda M, Resti Yektyastuti, Mahyumi Rantina, Nurul Hayati, Yudan Hermawan, Muhammad Adil Arnady

**Affiliations:** 1 Universitas Pendidikan Indonesia, Bandung, West Java, Indonesia; 2 Universitas Sriwijaya, Palembang, South Sumatra, Indonesia; 3 Universitas Sebelas Maret, Surakarta, Central Java, Indonesia; 4 Universitas Negeri Jakarta, East Jakarta, Special Capital Region of Jakarta, Indonesia; 5 Universitas Negeri Yogyakarta, Yogyakarta, Special Region of Yogyakarta, Indonesia

**Keywords:** Parental Perception, Children's Values, Family Dimensions, Location of Residence, Family Relationships.

## Abstract

This study investigated the relationship between parents perceptions of children’s values in Indonesian families, focusing on economic, social, emotional, and religious dimensions. The investigation was based on background factors, such as gender, location of residence, and education level, as well as correlations and regressions between dimensions of children’s values. The study participants consisted of 255 parents living in various urban and rural areas in Indonesia. Data were collected using the Children’s Values Scale adapted for the Indonesian context. The study findings showed that there were no significant differences in children’s perceptions of values based on gender or education level. However, there were significant differences based on location of residence, especially on economic and religious dimensions. Pearson correlation analysis revealed a significant positive relationship between social and emotional dimensions, while a negative relationship was found between economic and emotional dimensions in some contexts. The results of multiple linear regression analysis using the Stepwise method showed that economic and religious dimensions had a significant impact on overall family relationship patterns. This study highlights the importance of understanding the factors that influence parents’ perceptions of children’s values to support effective and harmonious parenting in Indonesian families.

## 1. Introduction

In every family, children have an important role both as individuals and as part of the overall family system. Children’s values are often perceived differently by parents, depending on the cultural, social, economic, and religious context ( Hoffman & Hoffman, 1973), ( Barni et al., 2019). These perceptions influence how parents educate their children and shape the overall family relationship pattern. Children’s values include aspects such as children as a source of emotional happiness, social support, economic investment, and inheritance of spiritual values ( Arnett, 2006). Understanding parents’ perceptions of children’s values is important because it contributes to the formation of children’s character and well-being, which has an impact on family dynamics ( Rahmawati et al., 2022).

Previous studies have shown that parents’ perceptions of their children’s values are influenced by factors such as socioeconomic status, education, and cultural experiences ( Santosa et al., 2023). Hoffman and Hoffman (1973) grouped children’s values into economic, social, and emotional dimensions, which reflect the child’s function in meeting the needs of parents. Ishak et al. (2016) highlighted that the religious dimension plays an important role in societies with strong religious traditions. However, many studies only explore one or two dimensions of children’s values, thus providing less comprehensive picture.

Research gaps are still visible, especially in the context of Indonesian society which is culturally diverse and has traditional values ( Putri et al., 2023). This study aims to explore the relationship between parents’ perceptions of children’s values from economic, social, emotional, and religious dimensions holistically. In addition, this study aims to understand its impact on family relationship patterns and child rearing. With a holistic approach, this study is expected to bridge the gap in previous research and provide new insights. The results of the study are expected to provide practical and theoretical benefits. Practically, these findings can be used by educators, family counselors, and policy makers to design programs that support the role of families in child care and education ( Sharma et al., 2013). Theoretically, this study can enrich the literature on children’s values in the multicultural context of Indonesia, supporting harmonious and sustainable family dynamics ( Hidayat & Pratama, 2023).

### 1.1 Theoretical background

Theoretically, social exchange theory can be used to explain the relationship between parental perceptions and children’s values. This theory states that relationships between individuals are based on the principle of profit and loss. In the context of children’s values, parents may view children as assets that provide benefits, both economically, socially, emotionally, and spiritually ( Hoffman & Hoffman, 1973). For example, the economic value of children is often more dominant in families with low socioeconomic status, where children are considered a source of labor or support for the family’s economy.

Previous studies have also shown that factors such as gender, place of residence, educational background, and socioeconomic status influence parents’ perceptions of their children’s values. A study by Kagitcibasi (2007) found that in collectivist societies such as Indonesia, children’s social and emotional values tend to be more emphasized than economic values. Conversely, in societies with a more individualistic orientation, children’s economic values tend to decline, while emotional values and individual achievements are more emphasized.

In the Indonesian context, research by Astuti (2015) shows that parents in rural areas tend to assess children from economic and religious aspects, while parents in urban areas emphasize more on education and self-development aspects. This difference is influenced by the residential environment and access to educational resources.

In addition, parents’ educational background also plays an important role in shaping their perception of children’s values. Parents with higher levels of education tend to see children as a long-term investment, especially in terms of education and career ( Rahmawati, 2019). On the other hand, parents with lower education emphasize more traditional values, such as obedience and direct economic support.

Not all studies support the view that children’s values are entirely determined by external factors such as socioeconomic status or educational attainment. Some studies, such as those, suggest that psychological factors, such as emotional satisfaction and parenting styles, are more dominant in shaping children’s perceptions of value than demographic factors ( Trumbell et al., 2018). This highlights the importance of understanding the subjective dimensions of parents’ perceptions of their children.

### 1.2 The present study

This study aims to investigate the relationship between parental perceptions of children’s values and various background factors in the context of families in Indonesia. The main focus of this study is to understand whether there are differences in parental perceptions of children’s values based on factors such as gender, place of residence, education level, and socioeconomic status. Place of residence is divided into urban and rural areas, with the assumption that the residential environment can influence parents’ views of children’s values. Educational background refers to the highest level of education achieved by parents, ranging from elementary education to higher education. Socioeconomic status is defined based on the family’s income level. Therefore, the following research questions are asked:

1. Do male and female parents differ in their view of children’s values in the family?
2. Is there a difference in the location of residence related to children’s values in the family?
3. Is there a difference in educational background and socioeconomic status related to children’s values in the family?
4. What is the relationship between the economic dimension and the social dimension, emotional dimension and religious dimension related to children’s values?

## 2. Materials and methods

### 2.1 Participants

The sample consisted of 255 parents of children aged 1-8 years across Indonesia. Data were collected through an online survey, and participation was completely voluntary. All participants provided written informed consent regarding their agreement to participate before submitting their responses. To ensure participant confidentiality, their identities were anonymized prior to analysis. Table 1 shows the demographic details of the parents who participated in the study.

**Table 1.** Parents’ demographic profile.

Karakteristik demografis		*f*	*(%)*
Gender	Female	205	80,4
	Male	50	19,6
Residence	City	70	27,5
	Village	185	72,5
Education_Background	Elementary School	6	2,4
	Junior High School	16	6,3
	Senior High School	82	32,2
	Diploma	28	11,0
	Bachelor	64	25,1
	Magister	52	20,4
	Doctor	7	2,7
Social_Economic_Status	Low	91	35,7
	Middle	99	38,8
	High	65	25,5

Note: *N* = 255, SD = Standard deviation.

**Ethical approval**

This study was conducted in accordance with the principles of the Declaration of Helsinki for research involving human participants. Ethical approval was obtained from the Ethics Committee of Universitas Pendidikan Indonesia (UPI), Bandung, Indonesia, under ethical clearance number B/EC/UPI/2023/067.

The committee confirmed that the study met national and institutional ethical standards for research involving human subjects. Given the non-interventional nature of the study, the use of anonymous self-reported data, and the absence of physical or psychological risk to participants, no additional ethical review was required beyond this approval.

**Informed consent**

Written Informed consent was obtained electronically from all participants prior to their participation in the study. Before accessing the questionnaire, participants were provided with detailed information regarding the study objectives, procedures, voluntary participation, confidentiality, and data use. Consent was indicated by participants actively selecting an agreement option before proceeding to the survey. As all participants were adults, no parental consent or assent procedures were required.

### 2.2 Instruments

The child value instrument used in this study was adapted from the work of Prof. Euis Sunarti, an expert in Family and Consumer Sciences at IPB University. This instrument is designed to measure various dimensions of children’s values, including economic, social, emotional, and religious aspects, which are relevant in the context of Indonesian families and culture ( Sunarti, 2020). The child value questionnaire uses a five-point Likert scale from 1 (very inappropriate) to 5 (very appropriate) with four dimensions.

Economic dimension (5 items) Measures the value of children as economic assets of the family, including financial contributions and economic support of children to parents, both directly and in the future. Social dimension (10 items) Describes the role of children in strengthening family social relationships, such as improving the social status of parents or expanding social networks through children’s interactions with the environment. Emotional dimension (5 items) refers to the value of children as a source of happiness, emotional closeness, and pride for parents, which creates deep emotional bonds within the family. Religious dimension (5 items) Measures the value of children as part of religious responsibilities, including parents’ expectations of children in carrying out religious values and becoming a source of pride in a spiritual context. There are 25 items in total, with good reliability of children’s values, with Cronbach’s alpha values ranging from 0.68 to 0.77 based on the reliability test.

In this study, both scales were translated using back-and-forth translation into Indonesian and English by two linguists and one education expert. A background questionnaire was added to collect information related to participants such as gender, age group, type of university, and teaching experience. The data were cleaned and entered into Statistical Package for the Social Sciences (SPSS) Software version 29 (IBM Corp, 2020). However, data from reversed items were recoded for analysis purposes.

### 2.3 Data collection procedures

The data collection procedure was conducted through an online invitation form distributed through the Family Activists group and WhatsApp group using random sampling. Ethical permission to use the questionnaire was granted by the Indonesian University of Education. Participants accessed the questionnaire through a link to the Platform, which outlined the purpose of the study and included an option for individuals to join the study voluntarily. This link can be opened using various internet browsers such as Firefox, Safari, Chrome, and others. Before starting the questionnaire, participants were required to read the instructions and provide their consent to participate in the study. After that, data containing person identification was coded to provide anonymous data, and all participant data was converted into an SPSS file.

### 2.4 Data analysis

SPSS version 29 (IBM Corp, 2020) was used to conduct descriptive and inferential statistics. Raw data from the online form with Likert scale were cleaned and transformed into SPSS dataset. Reliability was assessed using Cronbach’s alpha (α). Mean (M) by dimension, standard deviation, skewness, and kurtosis were also evaluated to provide descriptive information and ensure normality of the data. Descriptive statistics were used to describe the demographics of the participants. Inferential statistics, t-test with effect size using Cohen’s d ( Cohen, 2013), and one-way ANOVA were applied to assess group differences based on background variables. To investigate the relationships among the economic, social, emotional, and religious dimensions of children’s values, Pearson correlation analysis and multiple linear regression analysis using the stepwise method were conducted.

## 3. Results and discussion

### 3.1 Reliability analysis and data normality

The results of the reliability analysis were obtained by calculating the Cronbach’s alpha value (α) of all dimensions of the instrument. Table 2 presents the reliability results based on the scale and dimensions.

**Table 2.** Reliabilities and descriptive information used in the used instrument.

Scales	Dimensions	Cronbach’s alpha ( *α*)	*M*	SD	Skewness	Kurtosis
**Children’s Value**	Economy	0,56	13,04	2,57	-0,13	-0,19
	Social	0,77	33,89	3,52	-0,10	-0,84
	Emotional	0,68	14,91	2,45	-0,04	0,18
	Religious	0,53	17,37	1,62	-0,85	0,96
	**Instrument level**	0,63	19,80	2,54	-0,28	0,11

Note: *N* = 255, *M* = Mean, SD = Standard deviation.

The results of the reliability and descriptive tests showed that the scales used had varying levels of reliability, with the highest Cronbach’s alpha (α) value in the social dimension (α = 0.77) and the lowest in the religious dimension (α = 0.53). The highest average score was in the social dimension (M = 33.89, SD = 3.52) and the lowest in the economic dimension (M = 13.04, SD = 2.57). The skewness and kurtosis values showed a data distribution that was close to normal in all dimensions. These results are consistent with research showing that the social dimension tends to be more stable in measurement because of its association with social interactions that are more frequently observed consistently ( Santosa et al., 2022). However, the low reliability in the religious dimension may reflect the influence of high personal variations, such as intrinsic beliefs and spiritual experiences, which are difficult to measure consistently ( Rahmawati et al., 2023). This research supports the view that although measurement instruments have varying reliability, the social dimension is often a more measurable aspect than other, more subjective dimensions such as religiosity.

### 3.2 Gender differences

Regarding the first research question about possible gender differences among parents, a t-test was applied to compare all dimensions in children’s values. As shown in Table 3, no significant differences were found in all dimensions measured, although the mean scores showed that male parents scored higher than female parents on certain dimensions. This finding is in line with the study of Santosa et al. (2022), which stated that gender roles are not always a significant differentiating factor in viewing children’s values, especially in the context of a collectivist culture. However, this finding is different from the study of Rahmawati and Pratama (2023) in the context of urban society, which found that female parents tend to give higher values to social and emotional dimensions than male parents.

**Table 3.** Comparison of male and female parents.

Dimensions	Males		Females						95 % CI of Cohen’s *d*	
	*M*	SD	*M*	SD	*F*	*t*	*p*	Cohen’s *d*	Lower	Upper
Economy	2,76	0,61	2,57	0,48	5,79	-2,34	0,01	-0,36	-0,68	-0,05
Social	3,45	0,35	3,37	0,35	0,12	-1,41	0,72	-0,22	-0,53	0,08
Emotional	3,14	0,48	2,94	0,48	0,22	-2,67	0,63	-0,42	-0,73	-0,11
Religious	3,52	0,27	3,46	0,33	3,04	-1,08	0,08	-0,17	-0,48	0,13

Note: *N* = 255, *M* = Mean, SD = Standard deviation.

Significant differences were found in the economic and emotional dimensions.

In the religious dimension, there was no significant difference between male and female parents, supporting the view that religious values are more influenced by personal beliefs and family cultural norms than gender factors ( Hidayat & Pratama, 2023). Overall, these results reflect that despite variations in mean scores, gender is not a major determinant in parents’ perceptions of their children’s values.

### 3.3 Residence type differences

To answer the second research question, a t-test based on location of residence was used to determine whether there were differences between urban and rural areas in viewing children’s values. Table 4 shows that there are differences in children’s values between parents living in urban and rural areas, it was found that location of residence affects several dimensions of children’s values. Parents in urban areas tend to place higher values on the economic and social dimensions, which are influenced by wider access to resources and exposure to a modern lifestyle ( Rahmawati et al., 2022; Martiastuti, 2020). In contrast, parents in rural areas place more emphasis on the emotional and religious dimensions of children, which are influenced by collective culture and closer social interactions in their communities ( Hidayat & Pratama, 2023; Siti Muntiatul, 2023).

**Table 4.** Comparison by place of residence.

Dimensions	City		Village						95 % CI of Cohen’s *d*	
	*M*	SD	*M*	SD	*F*	*t*	*p*	Cohen’s *d*	Lower	Upper
Economy	2,73	0,53	2,56	0,49	0,78	2,42	0,37	0,34	0,06	0,61
Social	3,42	0,34	3,37	0,35	0,46	0,94	0,49	0,13	-0,14	0,40
Emotional	3,04	0,52	2,96	0,47	2,98	1,19	0,08	0,16	-0,10	0,44
Religious	3,48	0,35	3,47	0,31	0,37	0,40	0,54	0,05	-0,21	0,33

Note: *N* = 255, *M* = Mean, SD = Standard deviation, Statistically significant differences are shaded, *p* < 0.05.

This finding is in line with research by Fu & Mohamed Hashim (2024) ( Kong, 2024) who observed significant differences in the allocation of educational resources between urban and rural areas, and Putri et al. (2023), who showed that children’s character values were better maintained in rural areas. Overall, location of residence plays an important role in shaping parents’ views on children’s values, with urban areas being more oriented towards material achievement, while rural areas emphasize social relationships and spiritual values.

### 3.4 Education background and social economic status

Regarding the third research question, parents were divided into seven groups, based on educational background and socio-economic status, as presented in Table 1 above. The groups for educational background were elementary school, junior high school, senior high school, diploma, bachelor’s, master’s and doctoral, while the groups for socio-economic status were low, medium and high. Educational background, Economic Dimension [F (6, 254) = 3.41, p > .05], Social Dimension [F (6, 254) = 1.22, p > .05], Emotional Dimension [F (6, 254) = 1.04, p > .05], Religious Dimension [F (6, 254) = 0.43, p > .05]. Socioeconomic status, Economic Dimension [F (2, 254) = 2.84, p > .05], Social Dimension [F (6, 254) = 0.33, p > .05], Emotional Dimension [F (6, 254) = 1.15, p > .05], Religious Dimension [F (6, 254) = 1.12, p > .05]. The results of the one-way ANOVA test showed that educational background did not have a significant effect on the economic dimension [F (6, 254) = 3.41, p > 0.05], social [F (6, 254) = 1.22, p > 0.05], emotional [F (6, 254) = 1.04, p > 0.05], or religious [F (6, 254) = 0.43, p > 0.05]. Socioeconomic status also did not show a significant effect on the economic [F(2, 254) = 2.84, p > 0.05], social [F(6, 254) = 0.33, p > 0.05], emotional [F(6, 254) = 1.15, p > 0.05], or religious dimensions [F(6, 254) = 1.12, p > 0.05]. These results are consistent with recent research which states that education and socioeconomic status variables are not always the main determinants in shaping the economic, social, emotional, and religious aspects of individuals, especially in societies with collective cultural values ( Santoso et al., 2022). However, several studies have shown that education and socioeconomic status can play a significant role in the context of individualistic societies that are more oriented towards material achievement and social status ( Rahmawati & Pratama, 2023). Thus, these results support the view that intrinsic factors such as beliefs, cultural norms, and personal experiences can be more dominant in influencing social, emotional, and religious dimensions.

The results of the comparative analysis showed that there was a significant difference in the economic dimension between male parents (M = 2.76, SD = 0.61) and female parents (M = 2.57, SD = 0.48), with a t value = -2.34 (p < 0.05) and a moderate effect size (Cohen’s d = -0.36). In the emotional dimension, the difference was also significant with a higher average in men (M = 3.14, SD = 0.48) than women (M = 2.94, SD = 0.48), t = -2.67 (p < 0.05, Cohen’s d = -0.42). Meanwhile, no significant differences were found in the social and religious dimensions (p > 0.05). This finding is consistent with research showing that gender roles influence economic decisions and emotional stability, where men tend to have greater economic responsibilities ( Putri & Rahayu, 2021). However, the absence of significant differences in the social and religious dimensions supports the theory that these aspects are more influenced by universal family and cultural values ( Santosa et al., 2022). These results indicate that although there are gender differences in economic and emotional aspects, social and religious values remain relatively uniform across genders.

The results of the comparative analysis based on place of residence showed that there was a significant difference in the economic dimension between cities (M = 2.73, SD = 0.53) and villages (M = 2.56, SD = 0.49) with a t value = 2.42 (p < 0.05) and a moderate effect size (Cohen’s d = 0.34). The social, emotional, and religious dimensions did not show significant differences between cities and villages (p > 0.05). This finding is consistent with the latest theory stating that urban environments are more supportive of economic access and career development opportunities, which strengthens individual economic stability ( Rahmawati & Santoso, 2022). However, the insignificant differences in the social, emotional, and religious dimensions support the view that these factors are more influenced by interpersonal relationships and intrinsic beliefs that are independent of geographic location ( Hidayat & Pratama, 2023). Thus, although place of residence influences the economic aspect, its influence on the social, emotional, and religious dimensions is more evenly distributed across environments, indicating the importance of cultural factors and personal values in shaping these aspects.

### 3.5 Correlation and regression between dimensions of children’s values

Table 5 presents the Pearson correlation coefficients among the four dimensions of children’s values, illustrating the strength and direction of the relationships between economic, social, emotional, and religious dimensions.

**Table 5.** Pearson correlation between dimensions of children's values.

	ECO	SOS	EMO
ECO			
SOS	,360 ^**^		
EMO	,373 ^**^	,518 ^**^	
REG	,195 ^**^	,440 ^**^	,350 ^**^

** Correlation is significant at the 0.01 level (2-tailed).

Furthermore, the results of the multiple regression analysis are summarized in Table 6, showing the predictive role of the economic dimension on social, emotional, and religious dimensions.

**Table 6.** Regression analysis to predict how economic dimensions affect social dimensions, emotional dimensions and religious dimensions.

	Influence by	B	β	t	p(t)	R	R ^2^	Adj R ^2^	F	P(F)
Economic	Social	1.65	0.22	3.40	0.001	0.42	0.17	0.17	18.02	0,001
	Emotional	1.34	0.25	3.83	0.001					
	Religious	0.05	0.07	1.17	0.074					

Note: *B*: Unstandardized regression coefficient; *β*: standardized regression coefficient; statistically significant differences are shaded.

Pearson correlation analysis shows that the economic dimension has a significant relationship with the social (r = 0.360, p < 0.01) and emotional (r = 0.373, p < 0.01) dimensions, but the relationship with the religious dimension is weaker (r = 0.195, p < 0.01). This finding is in line with recent studies stating that socio-economic factors contribute significantly to mental health and individual social behavior ( Kusumawati, 2020). In addition, the economic dimension has been shown to strengthen emotional relationships through financial stability which improves psychological well-being ( Sari & Pratiwi, 2022). However, the relationship between economics and religiosity tends to be complex, because religiosity is more influenced by intrinsic values than materialistic factors ( Rahmawati et al., 2023). This study emphasizes that although economics influences social and emotional aspects, its influence on religiosity is indirect and requires an interdisciplinary approach to understand the factors that mediate the relationship. Thus, these results strengthen the view that the social and emotional dimensions are more influenced by economic stability compared to the religious dimension which is more tied to spiritual values and cultural context.

Based on the regression analysis, the economic dimension has a significant effect on the social and emotional dimensions, but not significant on the religious dimension. The economic dimension explains 17% of the variability in the social dimension (R ^2^ = 0.17). These results support research showing that economic status contributes to a person’s social and emotional behavior, especially through access to resources and increased well-being ( Alam et al., 2021). In addition, the significant economic dimension on the emotional aspect is in line with the theory of emotional well-being which is influenced by an individual’s economic stability ( Saraswati et al., 2020). However, the effect of the economic dimension on religiosity is not significant, reflecting the view that religious values are more often influenced by intrinsic factors such as personal beliefs than economic factors ( Hidayati & Fathoni, 2022). This suggests that although the economic dimension can increase social interaction and emotional well-being, the religious aspect is more influenced by spiritual and cultural experiences. Recent research confirms that religiosity has a weak relationship with economic well-being because the role of spirituality is more universal and does not depend on materialistic aspects ( Rahmawati et al., 2023). Thus, the economic influence on the social and emotional dimensions is more significant than its influence on the religious dimension.

## 4. Research implications

Investigating the relationship between parental perceptions of children’s values in Indonesian families has significant implications, particularly in the context of family dynamics and social policy. This study highlights the need to design more structured parenting programs to help parents understand the various dimensions of children’s values, such as economic, social, emotional, and religious. This can be achieved through family training and education designed to strengthen parents’ understanding of the importance of children’s values in building a harmonious family.

Educational institutions and communities can play an important role in facilitating interdisciplinary discussions and providing ongoing training opportunities for parents, especially in integrating traditional and modern values into their parenting practices. The Indonesian government can use the insights from this study to formulate social policies that support family well-being, such as providing access to educational resources and skills development programs for parents.

These policies could include incentives for communities that promote positive parenting values, as well as funding for programs designed to improve parents’ understanding of the child’s role in the family. Policymakers could also establish national standards for parenting programs that include economic, social, emotional, and religious dimensions as core competencies for families. In addition, further research funding is needed to explore effective strategies for building balanced and sustainable parenting that supports holistic child development and creates harmonious family dynamics.

## 5. Conclusion, limitations, and further developments

This study contributes to fill the gap in investigating the relationship between parental perceptions of children’s values in Indonesian families. In addition, background variables such as gender, location of residence, education level, and socioeconomic status are presented to enrich the results and discussions on children’s values in economic, social, emotional, and religious dimensions. The results showed that parents living in urban areas tend to have higher scores in economic and social dimensions, while parents in rural areas emphasize emotional and religious values more. Correlation analysis showed significant positive and negative relationships between these dimensions, while regression analysis revealed that economic and social dimensions have significant impacts on parents’ overall perceptions of children’s values.

However, this study has several limitations. First, the quantitative research design with a cross-sectional approach limits the ability to understand the dynamics of changes in parents’ perceptions over time. Longitudinal studies can provide deeper insights. Second, differences in sample sizes between groups based on location or education level make the generalizability of the findings more limited. Therefore, future research can involve more diverse participants and include other relevant factors, such as parenting experience.

Third, this study only used correlation and regression analysis due to the lack of previous studies comparing children’s values as latent factors in a more complex model. Future studies are expected to apply Structural Equation Modeling (SEM) to analyze the influence of confounding factors on children’s value perceptions in the family context. Thus, the findings of this study can provide new insights to support Indonesian families in understanding and applying children’s values holistically, which is useful for educational institutions, governments, and policy makers in developing better social policies and parenting programs.

## Ethical approval

This study was conducted in accordance with the principles of the Declaration of Helsinki for research involving human participants. Ethical approval was obtained from the Ethics Committee of Universitas Pendidikan Indonesia (UPI), Bandung, Indonesia, under ethical clearance number B/EC/UPI/2023/067.

The committee confirmed that the study met national and institutional ethical standards for research involving human subjects. Given the non-interventional nature of the study, the use of anonymous self-reported data, and the absence of physical or psychological risk to participants, no additional ethical review was required beyond this approval.

## Informed consent

Written Informed consent was obtained electronically from all participants prior to their participation in the study. Before accessing the questionnaire, participants were provided with detailed information regarding the study objectives, procedures, voluntary participation, confidentiality, and data use. Consent was indicated by participants actively selecting an agreement option before proceeding to the survey. As all participants were adults, no parental consent or assent procedures were required.

## Data Availability

The datasets generated and analyzed during the current study are not publicly available due to ethical and privacy restrictions, as they contain sensitive information related to family perceptions, parental values, and household contexts. The Ethics Committee of Universitas Pendidikan Indonesia (UPI) advised that unrestricted public sharing of the raw dataset could pose potential risks to participant confidentiality, even after anonymization. Therefore, the data are subject to controlled access. An anonymized version of the dataset may be made available upon reasonable request yantikarmila@upi.edu to the corresponding author. Requests will be reviewed on a case-by-case basis and access will be granted for academic purposes only, subject to ethical approval and a data use agreement that ensures confidentiality and non-identifiability of participants.

## References

[ref26] Alam MS Rahman MM Hossain MA : Economic well-being and social behavior: Evidence from family and community contexts. *Journal of Social and Economic Development.* 2021;23(2):345–360.

[ref1] Arnett JJ : Emerging Adulthood: Understanding the New Way of Coming of Age. Arnett JJ Tanner JL , editors. *Emerging adults in America: Coming of age in the 21st century.* American Psychological Association;2006; pp.3–19. 10.1037/11381-001

[ref2] Astuti D : Nilai anak dalam konteks pedesaan dan perkotaan di Indonesia. *Jurnal Sosiologi dan Pendidikan.* 2015;22(3):134–145.

[ref3] Barni D Ranieri S Ferrari L : Parents’ perceptions of their adolescent children’s personal values: Truth or bias? *J. Fam. Stud.* 2019;25(3):319–336. 10.1080/13229400.2016.1259120

[ref25] Cohen J : *Statistical power analysis for the behavioral sciences.* 2nd ed. New York, NY: Routledge;2013.

[ref4] Fu W Mohamed Hashim AT : Research on the Relationship between Urban and Rural Educational Resources Allocation and Educational Equity. *International Education Forum.* 2024;2(8):39–45. 10.26689/ief.v2i8.8329

[ref28] Hidayati N Fathoni A : Religiosity and value formation in family life: Evidence from Indonesian households. *Journal of Islamic Social Studies.* 2022;14(2):101–115.

[ref5] Hidayat M Pratama N : Rural parenting and the emphasis on emotional and religious values in children. *Journal of Rural and Community Development.* 2023;18(2):89–102.

[ref6] Hoffman LW Hoffman ML : The value of children to parents. Fawcett JT , editor. *Psychological perspectives on population.* Basic Books;1973; pp.45–70.

[ref8] Ishak M : Religious influences in parenting values. *Journal of Behavioral and Cultural Studies.* 2016;22(3):45–60.

[ref9] Kagitcibasi C : *Family, self, and human development across cultures: Theory and applications.* Lawrence Erlbaum Associates; 2nd ed. 2007.

[ref10] Kong C : Urban-rural differences in the impact of family educational inputs on adolescent mental health. *Interdisciplinary Humanities and Communication Studies.* 2024;1(8). 10.61173/twm21a89

[ref11] Kusumawati D : Pengaruh faktor sosial ekonomi terhadap perilaku sosial masyarakat. *Jurnal Ilmu Sosial.* 2020.

[ref12] Martiastuti N : Parental perceptions of child values. *Journal of Educational Studies.* 2020;15(2):45–60.

[ref13] Muntiatul S : Environmental influences on child character development. Kompasiana. 2023. Reference Source

[ref14] Putri F Rahayu N : Gender roles in economic decision-making. *Journal of Family Economics.* 2021.

[ref15] Putri F : Character values in rural and urban contexts. *J. Soc. Psychol.* 2023;29(1):32–48.

[ref16] Rahmawati T : Pendidikan orang tua dan pengaruhnya terhadap persepsi nilai anak. *Jurnal Pendidikan dan Keluarga.* 2019;11(2):89–101.

[ref17] Rahmawati T : Urban vs rural perspectives on child value dimensions. *Journal of Family and Social Studies.* 2022;35(3):134–145.

[ref18] Rahmawati T : Religiusitas dan konteks sosial ekonomi. *Jurnal Studi Agama.* 2023.

[ref19] Santosa D : Cultural influences on social and religious dimensions. *J. Cult. Stud.* 2022.

[ref20] Santosa D : Social interactions and child value in urban settings. *Journal of Urban Sociology.* 2023;28(4):210–225.

[ref27] Saraswati N Putri RA Lestari D : Emotional well-being and family stability: A psychosocial perspective. *Journal of Applied Psychology and Family Studies.* 2020;12(1):45–58.

[ref21] Sari M Pratiwi N : Kesejahteraan psikologis dan stabilitas ekonomi. *Jurnal Psikologi Terapan.* 2022.

[ref22] Sharma U : Teachers’ attitudes towards inclusive education. *Int. J. Incl. Educ.* 2013;17(1):15–29.

[ref23] Sunarti E : Instrumen penilaian nilai anak dalam konteks keluarga Indonesia. *Jurnal Ilmu Keluarga dan Konsumen, IPB University.* 2020.

[ref24] Trumbell JM Hibel LC Mercado E : The impact of marital withdrawal and secure base script knowledge on mothers’ and fathers’ parenting. *J. Fam. Psychol.* 2018;32(6):699–709. 10.1037/fam0000402 29927289 PMC6126982

