# Supplementary material for: How Parents Perceive Children’s Values in Indonesian Families: A Multidimensional and Contextual Analysis
Source: F1000Res. 2026 Mar 26;15:106. Originally published 2026 Jan 23. [Version 2] doi: 10.12688/f1000research.176108.2 (PMC13122144; doi:10.12688/f1000research.176108.2)
Supplement: Peer review history

# Reviewer report: Reviewer response for version 2

**DOI:** 10.5256/f1000research.197535.r471408

**Published:** 2026-04-27

**Authors:** Tesya Cahyani Kusuma

**Affiliations:** 1 Universitas Adzkia, Padang, Indonesia

This study examines parents’ perceptions of children’s values in Indonesian families using a multidimensional framework (economic, social, emotional, and religious dimensions). The study employs a quantitative cross-sectional design with 255 participants and applies various statistical analyses, including t-tests, ANOVA, correlation, and regression. The findings highlight the role of residential context in shaping parental perceptions, while gender and educational background appear to have limited influence. Overall, the manuscript provides a relevant contribution to the literature on family studies in the Indonesian context.

**Major Comments (Minor–Moderate Revision)**

1. **Clarity of Sampling Method**
  The manuscript states that random sampling was used; however, data collection was conducted via online distribution (e.g., WhatsApp groups), which more closely resembles convenience sampling. The authors are encouraged to clarify the sampling technique to improve methodological transparency.
2. **Consistency in Statistical Interpretation**
  There are some inconsistencies between the reported statistical results and their interpretation. For example, certain sections state that no significant differences were found, whereas tables indicate statistically significant results (e.g., gender differences in economic and emotional dimensions). This should be carefully reviewed and revised for consistency.
3. **Justification of Statistical Approach**
  The use of stepwise regression analysis should be briefly justified, as this method has known limitations. A short explanation would strengthen the methodological rigor of the study.
4. **Instrument Adaptation and Validation**
  While the manuscript mentions translation procedures, further clarification on the adaptation and validation process of the instrument in the Indonesian context would enhance reproducibility and credibility.

**Minor Comments**

1. **Literature Integration**
  Some parts of the Introduction and Discussion remain descriptive. The manuscript would benefit from a more critical synthesis of the literature to better highlight the research gap and contribution.
2. **Data Availability Statement**
  The explanation regarding restricted data access is understandable. However, the authors may consider providing more detail on the conditions under which data can be accessed (e.g., type of request, approval process).
3. **Clarity of Writing**
  Overall, the manuscript is well written. Minor language polishing may further improve readability, particularly in linking results with interpretations.    

**Conclusion**

 The conclusions are generally supported by the results and are presented in a balanced and cautious manner. The manuscript offers useful insights and has potential relevance for research, practice, and policy in family and parenting contexts.

Is the work clearly and accurately presented and does it cite the current literature?

Partly

If applicable, is the statistical analysis and its interpretation appropriate?

Partly

Are all the source data underlying the results available to ensure full reproducibility?

Partly

Is the study design appropriate and is the work technically sound?

Yes

Are the conclusions drawn adequately supported by the results?

Yes

Are sufficient details of methods and analysis provided to allow replication by others?

Partly

Reviewer Expertise:

Early Childhood Critical Thinking, Culturally Responsive Pedagogy, Society 5.0 & Future Skills.

I confirm that I have read this submission and believe that I have an appropriate level of expertise to confirm that it is of an acceptable scientific standard.

# Reviewer report: Reviewer response for version 2

**DOI:** 10.5256/f1000research.197535.r470977

**Published:** 2026-04-10

**Authors:** Andi Hasdiansyah

**Affiliations:** 1 Universitas Muhammadiyah Parepare, Parepare, Indonesia

The authors have addressed all my previous concerns and suggestions effectively. I am satisfied with the revisions made to the manuscript, and I have no further comments. The article is now suitable for indexing.

Is the work clearly and accurately presented and does it cite the current literature?

Yes

If applicable, is the statistical analysis and its interpretation appropriate?

Yes

Are all the source data underlying the results available to ensure full reproducibility?

Partly

Is the study design appropriate and is the work technically sound?

Yes

Are the conclusions drawn adequately supported by the results?

Yes

Are sufficient details of methods and analysis provided to allow replication by others?

Partly

Reviewer Expertise:

Nonformal education, community empowerment, ecopedagogy, social development

I confirm that I have read this submission and believe that I have an appropriate level of expertise to confirm that it is of an acceptable scientific standard.

# Reviewer report: Reviewer response for version 1

**DOI:** 10.5256/f1000research.194140.r461595

**Published:** 2026-02-28

**Authors:** Andi Hasdiansyah

**Affiliations:** 1 Universitas Muhammadiyah Parepare, Parepare, Indonesia

**Summary of the Article**This article investigates parents' perceptions of children's values in Indonesian families through a multidimensional (economic, social, emotional, and religious) and contextual analysis (gender, residential location, education level, and socioeconomic status). Using a sample of 255 parents, the study found that residential location (urban vs. rural) is a significant factor influencing the perception of economic and religious values, while gender and education did not show significant differences.

**Article Evaluation**

***Clarity, Accuracy, and Current Literature***

 The work is presented with a clear and logical structure, adhering to international research article standards. The title accurately reflects the content of the research. The authors have cited highly current literature (2020-2024), including key references from the local Indonesian context (such as Sunarti, Rahmawati, and Hidayat) as well as international theories (e.g., Social Exchange Theory). This demonstrates a good understanding of the current research landscape.

***Research Design and Academic Value***

 The quantitative cross-sectional research design employed is appropriate for exploring relationships between variables. This study holds significant academic value as it addresses a gap in the literature regarding children's value perceptions in Indonesia using a holistic approach that combines four dimensions simultaneously, which is rarely done in previous studies that tend to focus on only one or two dimensions.

***Methods and Potential for Replication***

 The detailed methods provided are sufficient to allow for replication. The authors describe:

 1. Detailed sample characteristics (Table 1).

 2. The instrument used (an adaptation of Prof. Euis Sunarti's scale), including the number of items and measurement scale.

 3. Data collection procedures via online survey.

 4. Data analysis steps using SPSS. Suggestion: The authors are encouraged to include the complete instrument (questionnaire) as supplementary material to enhance transparency and ease of replication.

***Statistical Analysis and Interpretation***

 The statistical analyses used (t-test, ANOVA, Pearson correlation, and multiple linear regression) are appropriate for answering the research questions. However, there are critical notes on the following aspects:

 1. Instrument Reliability: The Cronbach’s Alpha values for the Religious (0.53) and Economic (0.56) dimensions are below the common thresholds (0.60 or 0.70). While the authors acknowledge this in the discussion, the interpretation of results for these dimensions should be done with greater caution.

 2. Sample Imbalance: The sample is dominated by females (80.4%) and rural residents (72.5%). Although the authors discuss this limitation, the generalization of results should be limited to these sample characteristics.

 - Data Availability and Reproducibility

 The authors state that the raw data are not publicly available due to ethical and privacy concerns related to sensitive family information. However, they provide an access mechanism through requests to the corresponding author. This is acceptable in the context of sensitive social research.

 - Conclusions and Support for Findings

 The conclusions drawn are adequately supported by the data analysis results. Findings regarding differences in perceptions based on residential location are consistent with the data presented in Table 4. The authors also honestly state the limitations of the study, which adds to the credibility of the work.

 1. Suggestions for Authors:

 - Instrument Validity: Provide a more in-depth explanation of why the reliability for the religious and economic dimensions is low. Are there specific items that might be ambiguous in the current Indonesian context?

 - Discussion of Implications: Strengthen the policy implications section with more specific recommendations for parenting programs in Indonesia based on the urban/rural differences found.

 - Appendices: If possible, include the research instrument in both Indonesian and its English translation to assist other researchers.

Is the work clearly and accurately presented and does it cite the current literature?

Yes

If applicable, is the statistical analysis and its interpretation appropriate?

Yes

Are all the source data underlying the results available to ensure full reproducibility?

Partly

Is the study design appropriate and is the work technically sound?

Yes

Are the conclusions drawn adequately supported by the results?

Yes

Are sufficient details of methods and analysis provided to allow replication by others?

Partly

Reviewer Expertise:

Nonformal education, community empowerment, ecopedagogy, social development

I confirm that I have read this submission and believe that I have an appropriate level of expertise to confirm that it is of an acceptable scientific standard, however I have significant reservations, as outlined above.

# Response

**Published:** 2026-03-02

**Authors:** Yanti Karmila Nengsih

**Affiliations:** Community Education, Universitas Pendidikan Indonesia, Bandung, West Java, Indonesia

We sincerely thank the reviewer for the thorough, constructive, and insightful evaluation of our manuscript. We greatly appreciate the positive assessment regarding the clarity, structure, methodological rigor, and academic contribution of our study. Below, we provide point by point responses to the reviewer’s comments.

 1. We intentionally structured the manuscript following international research article standards and prioritized recent literature while integrating local Indonesian scholarship to ensure contextual relevance. No revisions were made to this section as it already meets the intended clarity and academic rigor.

 2. The multidimensional approach (economic, social, emotional, and religious) was deliberately adopted to address limitations in previous studies that focused on only one or two dimensions. We believe this holistic framework strengthens the originality and academic value of the study.

 3. The instrument used in this study is an adapted version of the Children’s Values Scale developed by Prof. Euis Sunarti. Due to intellectual property considerations and ethical agreements, we are currently unable to publish the full questionnaire publicly. However, we have provided a detailed description of each dimension, the number of items, scaling method, and reliability indices to ensure transparency and replicability. We have clarified this point in the Methods section.

 4. Discussion sections, the lower reliability coefficients likely reflect the context-sensitive and subjective nature of economic and religious values in Indonesian families, which are influenced by diverse cultural, spiritual, and socioeconomic interpretations. These dimensions capture complex constructs that are inherently more variable. We have emphasized caution in interpreting findings related to these dimensions and reinforced this explanation in the Discussion section.

 5. This limitation has been explicitly acknowledged in the Limitations section. The imbalance reflects the voluntary nature of online survey participation and the higher engagement of mothers in parenting related research. Consequently, we have limited the scope of generalization to populations with similar demographic characteristics.

 6. As stated in the Data Availability section, the dataset contains sensitive family-related information. Controlled access through the corresponding author was implemented in accordance with ethical approval guidelines, ensuring both participant confidentiality and research transparency.

 7. The policy implications section has been strengthened by explicitly linking the findings particularly urban rural differences to the development of targeted parenting education programs and community based family interventions in Indonesia. These additions aim to enhance the practical relevance of the study.

 8. We acknowledge the value of this recommendation. While full publication of the instrument is constrained by ethical and intellectual property considerations, we have enhanced the instrument description and clarified the adaptation and translation procedures to support future research efforts.

 Once again, we sincerely thank the reviewer for their constructive feedback, which has significantly strengthened the clarity, transparency, and overall quality of this manuscript.
